# Surveillance for Distant Metastasis in Breast Cancer Patients Who Underwent Contemporary Management: A Report from the Korean Breast Cancer Society Survivor Research Group

**DOI:** 10.1245/s10434-024-15665-3

**Published:** 2024-07-05

**Authors:** Jong-Ho Cheun, Sooyeon Chung, Jai Hong Han, Young-Won Lee, Ji-Jung Jung, Jung Whan Chun, Eun-Gyeong Lee, Jun Won Min, Zisun Kim, Jihyoun Lee, So-Youn Jung, Yoo Seok Kim, Jong Han Yu, Eun-Kyu Kim, Jong-Won Lee, Ki-Tae Hwang, Ku Sang Kim, Hyun Jo Youn, Hyeong-Gon Moon

**Affiliations:** 1 Department of Surgery, Seoul Metropolitan Government Seoul National University Boramae Medical Center, Seoul National University College of Medicine, Seoul, South Korea; 2 Department of Surgery, Samsung Medical Center, Sungkyunkwan University College of Medicine, Seoul, South Korea; 3 Center for Breast Cancer, National Cancer Center, Goyang, South Korea; 4 Department of Surgery, Asan Medical Center, Ulsan University College of Medicine, Seoul, South Korea; 5 Department of Surgery, Seoul National University Hospital, Seoul National University College of Medicine, Seoul, South Korea; 6 Department of Surgery, Korea University Anam Hospital, Korea University College of Medicine, Seoul, South Korea; 7 Department of Surgery, Dankook University Hospital, Dankook University College of Medicine, Cheonan, South Korea; 8 Department of Surgery, Soonchunhyang University Bucheon Hospital, Soonchunhyang University College of Medicine, Bucheon, South Korea; 9 Department of Surgery, Soonchunhyang University Seoul Hospital, Soonchunhyang University College of Medicine, Seoul, South Korea; 10 Department of Surgery, Chosun University Hospital, Chosun University College of Medicine, Gwangju, South Korea; 11 Department of Surgery, Seoul National University Bundang Hospital, Seoul National University College of Medicine, Seongnam, South Korea; 12 Department of Surgery, Gospel Hospital, Kosin University College of Medicine, Busan, South Korea; 13 Department of Surgery, Jeonbuk National University Hospital, Jeonbuk National University Medical School, Jeonju, South Korea

**Keywords:** Breast cancer, Surveillance, Imaging study, Distant metastasis, Mortality

## Abstract

**Background:**

Current guidelines recommend against the use of routine imaging tests to detect distant metastasis in asymptomatic breast cancer patients. However, recent advancements in effective therapeutics and diagnostic accuracy have raised the need to reassess the clinical efficacy of intensive metastasis surveillance. We report the results of a multicenter retrospective study to investigate the association between intensive imaging studies and survival outcomes.

**Patients and Methods:**

We retrospectively reviewed the data of 4130 patients who underwent surgery from 11 hospitals in Korea between January 2010 and December 2011. Patients were divided into two groups on the basis of the intensity of metastasis imaging studies during their disease-free period. The types and intervals of the imaging studies were based on each physician’s decisions.

**Results:**

High-intensive screening showed a shorter distant metastasis-free survival [*p* < 0.001, hazard ratio (HR) 1.62; 95% confidence interval (CI) 1.29–2.04], especially for patients in whom bone or lung was the first site of metastasis. With a median follow-up period of 110.0 months, the 5-year breast cancer-specific survival (BCSS) rate was 96.5%. The high-intensity screening group showed significantly poorer BCSS compared with the low-intensity screening group (*p* < 0.001, HR 3.13; 95% CI 2.32–4.21). However, both multivariable analysis and propensity score matching analysis showed no significant association between the screening intensity and BCSS.

**Conclusions:**

Frequent imaging studies to detect distant metastasis were associated with earlier detection of distant metastasis, especially for lung and bone metastasis. However, intensive surveillance showed no apparent association with BCSS despite the use of currently available treatments.

**Supplementary Information:**

The online version contains supplementary material available at 10.1245/s10434-024-15665-3.

The incidence of breast cancer has substantially increased worldwide during the last few decades, and it is also currently the most common female malignancy in Korea.^1,2^ As the overall survival rate for patients with breast cancer has steadily increased over time, a large number of breast cancer survivors are undergoing regular follow-up visits.^3,4^ The estimated number of breast cancer survivors in the USA was more than 4 million women in 2022.^5^

Current guidelines recommend against using imaging or laboratory tests to detect distant metastasis in asymptomatic patients with breast cancer after their initial treatment.^6,7^ These recommendations are based on the results of randomized clinical trials that showed no survival improvement with the use of intensive surveillance for distant metastasis.^8,9^ However, as acknowledged in the guidelines, there is an urgent clinical need to re-assess this issue since previous trials were conducted two decades ago when diagnostic tools were less accurate and therapeutics were less effective.^6^

Despite the current recommendations, real-world data show that breast cancer patients regularly undergo one or more imaging or laboratory tests to screen for distant metastasis.^10–12^ The frequent use of tests may also arise from the belief that earlier therapeutic intervention for distant metastasis may lead to survival benefits, based on the studies that have reported successful new therapeutics in metastatic breast cancer patients.^13–16^

In this study, we report the results of a multicenter retrospective study conducted by the Korean Breast Cancer Society Survivor Research Group (KBCS-SRG) that aimed to investigate the relationship between the use of frequent tests and survival for patients with breast cancer. We collected the real-world data of patients with breast cancer treated at 11 teaching hospitals in Korea during the period when subtype-based therapeutics, such as aromatase inhibitors or trastuzumab, were all approved for clinical use.

## Patients and Methods

### Patients

We retrospectively reviewed the clinicopathologic records of 4130 patients who underwent surgery for breast cancer between January 2010 and December 2011 from the 11 hospitals in South Korea. Patients with carcinoma in situ lesions, male breast cancer, recurrent breast cancer, metastatic breast cancer, and synchronous or metachronous malignancies in other organs were excluded. There were no restrictions on patients’ ages. Initial breast cancer was staged according to the American Joint Committee on Cancer (AJCC) cancer staging manual, the eighth edition.^17^ The information on the hormonal receptor status was collected as one parameter rather than separately for estrogen receptor and progesterone receptor. Therefore, patient classification was only possible by the anatomic staging system. Ki-67 expression level was categorized as high or low on the basis of each institution’s cutoff value. Positive hormone receptor (estrogen and/or progesterone receptors) was defined as ≥ 1% of stained cells or Allred scores above 2 on the immunohistochemistry assay. Human epidermal growth factor receptor type 2 (HER2) was evaluated with anti-HER2 antibodies, and patients with equivocal results underwent fluorescence in situ hybridization or silver-enhanced in situ hybridization tests. Among patients with HER2-positive breast cancer, those with tumor size of > 1.0 cm or metastatic axillary lymph nodes were treated with the HER2-targeted therapy according to the insurance coverage indications of the Korean National Health Insurance. High-volume centers were defined as those treating > 500 patients with breast cancer annually. The study was approved by the institutional review board of each institution. All procedures were conducted following the Declaration of Helsinki, and the requirement for informed consent was waived.^18^

### Intensity of Surveillance Exams

We gathered the data on the number of imaging tests conducted during the distant metastasis-free survival period after surgery. The use of various imaging tests depended on each physician’s consideration. The imaging modalities included bone scans, chest computed tomography (CT), abdomen ultrasonography (USG), and abdomen CT, all of which are commonly performed tests for surveillance in Korea.^10^ Imaging tests that were performed for symptoms unrelated to breast cancer were also included. Laboratory tests and plain X-rays were excluded in our analysis as these tests show low sensitivity to detect distant metastasis compared with other tests.

Patients were divided into two groups on the basis of the median value of measured screening intensity, calculated by dividing the total number of exams by the distant metastasis-free survival (DMFS) interval. We excluded ^18^Fludeoxyglucose-positron emission tomography (^18^F-FDG/PET) from our study to minimize selection bias, as the Korean National Health Insurance covers it only when distant metastasis is suspected on other imaging modalities.

### Definition of Recurrence and Survival Period

Locoregional recurrence events included ipsilateral breast tumor recurrence and recurrence at the ipsilateral chest wall, mastectomy scar, and regional lymph nodes, such as ipsilateral axillary, supra-/infra-clavicular, or internal mammary lymph nodes. Distant metastasis referred to any recurrence beyond the locoregional recurrence, such as metastasis to bone, lung, liver, and distant lymph nodes.

DMFS was defined as the interval between the date of surgery and the date of histologic confirmation of distant metastasis or the date of the last follow-up. When the biopsy was not feasible, DMFS was determined as the period until the date of clinical diagnosis that led to the initiation of palliative treatments. Breast cancer-specific survival (BCSS) was defined as the time from surgery to the death from breast cancer or breast cancer treatment-related complications.

### Statistics

The continuous variables were analyzed with the Mann–Whitney *U* test, and categorical variables were analyzed with Pearson’s *χ*^2^ test. The Log-rank test was used to compare the survival curves derived from the Kaplan–Meier method. Cox proportional hazards regression model was used for multivariable analysis and to estimate the adjusted hazard ratio. Variables with a two-sided *p* < 0.10 in the univariate analysis were analyzed in the multivariable analysis, and those that showed multicollinearity were excluded. Missing data were treated using a complete-case analysis approach. Statistical significance was set at *p* < 0.05. All analyses were conducted using SPSS, version 27.0 (SPSS, INC., IBM, Armonk, NY, USA), and the Kaplan–Meier curves were drawn using GraphPad Prism^TM^, version 9.0 (GraphPad Software, San Diego, CA, USA).

The propensity scores were estimated using a logistic regression model. Nearest-neighbor matching method was used for 1:1 matching without replacement using a caliper width of 0.10 standard deviation of the logit of the propensity scores. Variables that were differently distributed between the two groups were included in matching analysis. Propensity score matching was performed using the “MatchIt” R package (version 3.6.3).

## Results

### Patients

A total of 4130 patients with breast cancer who met the inclusion criteria were included in the study. Table 1 shows the clinicopathologic characteristics of the subjects and the use of imaging tests in these patients. As presented in Table 1, more than half of the patients had T1 (54.7%) and/or N0 (62.1%) status, and 3038 (73.6%) patients were treated with breast conservation surgery. The median frequency of surveillance during the DMFS period was 1.38 exams per year [interquartile range (IQR): 0.77–2.27]. During the median follow-up of 110 months, 175 (4.2%) patients experienced locoregional recurrence: 26 (14.9%) patients had distant metastasis concurrently and 33 (18.9%) patients developed distant metastasis later. A total of 301 (7.3%) patients developed distant metastasis during the follow-up period, among which 242 patients had no locoregional recurrence history. At the time of diagnosis of metastasis, 76 (25.2%) patients experienced symptoms related to distant metastasis.

**Table 1:** Demographic and clinicopathological characteristics of patients

	All patients (*n* = 4130)
Age at operation (years)	50.0 (44.0–56.0)
BMI (kg/m^2^)	23.2 (21.3–25.6)
Institutions	
High-volume center	3187 (77.2%)
Low-volume center	943 (22.8%)
Breast operation	
Breast conservation	3038 (73.6%)
Mastectomy	1092 (26.4%)
Axilla operation	
Sentinel lymph node biopsy or omitted	2351 (56.9%)
Axillary lymph node dissection	1779 (43.1%)
T stage ^a^	
T1	2258 (54.7%)
T2	1601 (38.8%)
T3-4	265 (6.4%)
Unknown	6 (0.1%)
N stage^a^	
N0	2565 (62.1%)
N1	1064 (25.8%)
N2	295 (7.1%)
N3	195 (4.7%)
Unknown	11 (0.3%)
Histologic grade	
I–II	2389 (57.8%)
III	1457 (35.3%)
Unknown	284 (6.9%)
Lymphovascular invasion	
Present	1290 (31.2%)
Absent	2688 (65.1%)
Unknown	152 (3.7%)
Hormone receptor status	
Positive	3109 (75.3%)
Negative	1017 (24.6%)
Unknown	4 (0.1%)
HER2 receptor status	
Positive	890 (20.8%)
Negative	3247 (78.6%)
Unknown	23 (0.6%)
Ki-67 index ^b^	
High	1489 (36.1%)
Low	2349 (56.9%)
Unknown	292 (7.1%)
Neoadjuvant chemotherapy	
Administered	327 (7.9%)
Not administered	3626 (87.8%)
Unknown	177 (4.3%)
Adjuvant chemotherapy	
Administered	2929 (70.9%)
Not administered	1182 (28.6%)
Unknown	19 (0.5%)
Adjuvant radiotherapy	
Administered	3283 (79.5%)
Not administered	824 (20.0%)
Unknown	23 (0.6%)
Adjuvant hormonal treatment	
Administered	3075 (74.5%)
Not administered	1039 (25.2%)
Unknown	16 (0.4%)
HER2-targeted treatment	
Administered	619 (15.0%)
Not administered	3406 (82.5%)
Unknown	105 (2.5%)
Locoregional recurrence	
Present	175 (4.2%)
Absent	3955 (95.8%)
Distant metastasis	
Absent	3829 (92.7%)
Bone	69 (1.7%)
Visceral single organ	133 (3.2%)
Multiple organs	99 (2.4%)
Intensity of imaging modalities (per year)	
Bone scan	0.7 (0.4–1.0)
Chest CT	0.4 (0.1–0.7)
Abdomen sonography	0.0 (0.0–0.5)
Abdomen CT	0.0 (0.0–0.1)

Number of patients (%) or median (IQR)

Data on patients who underwent breast cancer surgery between January 2010 and December 2011 from the 11 hospitals in South Korea were retrospectively reviewed

*BMI* body mass index, *HER2* human epidermal growth factor receptor-2, *CT* computed tomography

^a^Stratified according to the American Joint Committee on Cancer (AJCC) eighth TNM anatomic stage

^b^Stratified according to each institution’s criteria

### Surveillance Intensity and DMFS

**Fig. 1:**
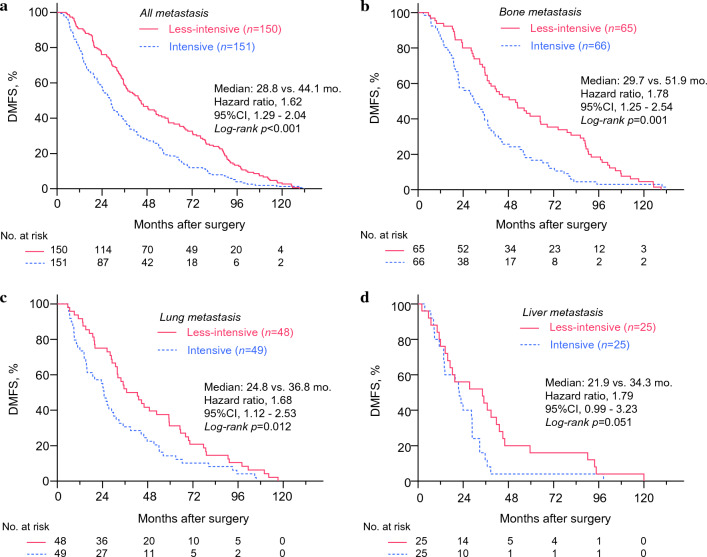
Kaplan–Meier curves showing distant metastasis-free survival among patients who developed distant metastasis. Patients who experienced distant metastasis after breast cancer surgery at the 11 hospitals in South Korea were retrospectively analyzed. The Kaplan–Meier curves show DMFS for 301 patients who eventually developed distant metastasis (**a**) and patients with bone (**b**), lung (**c**), and liver metastasis (**d**). *p* values were calculated by using the log-rank test, and the hazard ratio was calculated with univariate Cox-regression analysis. *DMFS* distant metastasis-free survival, *CI* confidence interval

We estimated the association between the intensive metastasis screening and time to distant metastasis by using the survival data of the 301 patients who eventually developed distant metastasis during the follow-up. As expected, highly intensive surveillance for distant metastasis showed significantly shorter distant metastasis-free survival among the 301 patients [*p* < 0.001, hazard ratio (HR) 1.62; confidence interval (CI) 1.29–2.04; Fig. 1a]. The difference in median time to distant metastasis between the two groups was 15.3 months (28.8 months for intensive surveillance vs. 44.1 months for less-intensive surveillance). We also observed that the initial site of metastasis was associated with the degree of lead-time. As shown in Fig. 1b–d, bone metastasis showed the most prolonged difference in median DMFS for metastasis surveillance (30.0 vs. 52.0 months, *p* <0.001), followed by lung metastasis (24.9 vs. 39.7 months, *p* = 0.004). Intensive metastasis surveillance was not significantly associated with DMFS in patients who developed liver metastasis (median 21.9 vs. 34.3 months, *p* = 0.051).

### Surveillance Intensity and BCSS

**Fig. 2:**
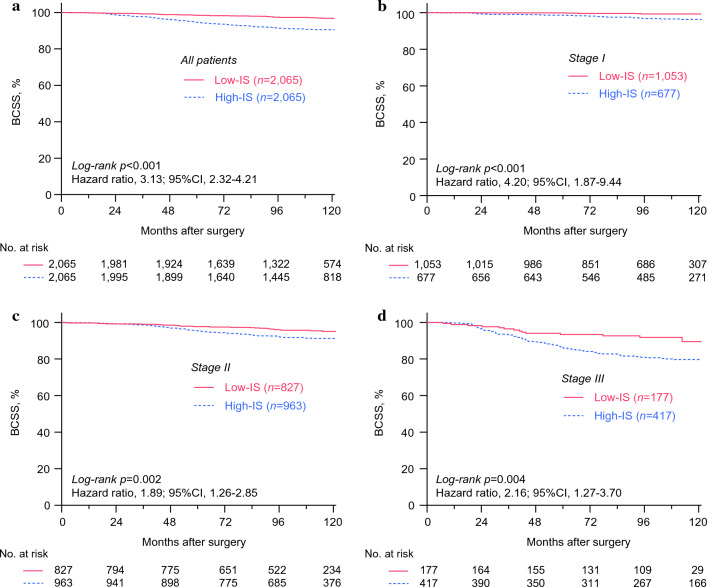
Kaplan–Meier curves showing breast cancer-specific survival. Data on patients who underwent breast cancer surgery between January 2010 and December 2011 at the 11 hospitals in South Korea were retrospectively analyzed. The Kaplan–Meier curves show BCCS for all patients (**a**) and patients with each anatomic stage (**b**–**d**) according to the intensity of surveillance. *p* values were calculated by using the log-rank test, and the hazard ratio was calculated with univariate Cox-regression analysis. *BCCS* breast cancer-specific survival, *Low-IS* low-intensity surveillance, *High-IS* high-intensity surveillance, *CI* confidence interval

**Fig. 3:**
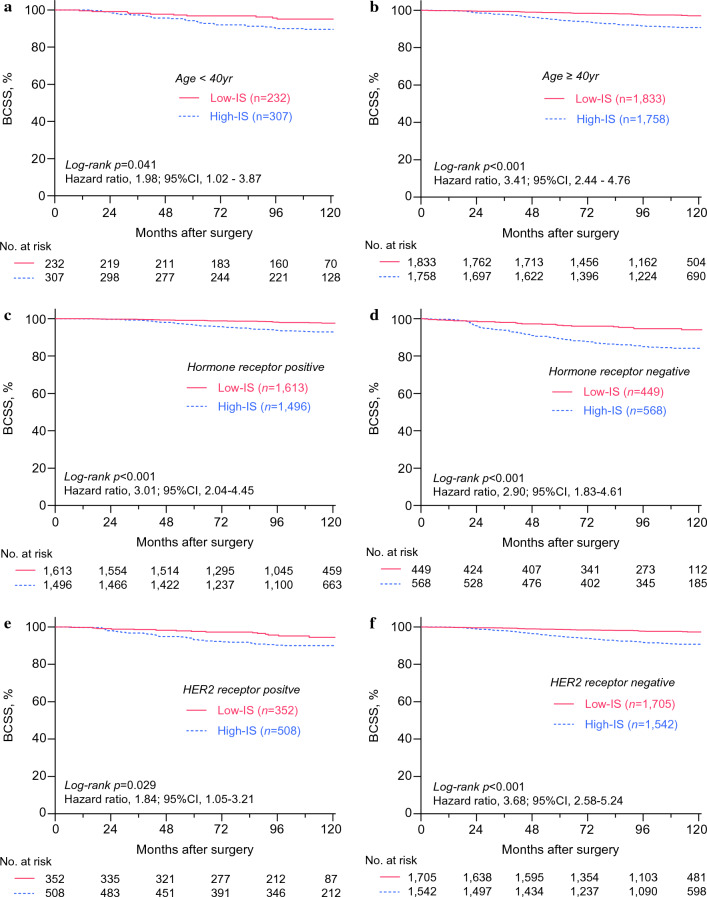
Kaplan–Meier curves showing breast cancer-specific survival according to age and tumor subtypes. Data on patients who underwent breast cancer surgery between January 2010 and December 2011 at the 11 hospitals in South Korea were retrospectively analyzed. The Kaplan–Meier curves show BCSS after stratification according to age at the surgery (**a**–**b**), hormone receptor (**c**–**d**), and HER2 receptor (**e**–**f**). *p* values were calculated by using the log-rank test, and the hazard ratio was calculated with univariate Cox-regression analysis. *BCCS* breast cancer-specific survival, *Low-IS* low-intensity surveillance, *High-IS* high-intensity surveillance, *CI* confidence interval, *HER2* human epidermal growth factor receptor type 2

Next, the whole study population (*n* = 4130) was grouped into the high-intensity surveillance (High-IS) or the low-intensity surveillance (Low-IS) groups on the basis of the median value of the screening frequency of all patients (Supplementary Fig. 1). The median frequency of the tests was 2.26 (IQR, 1.81–3.12) and 0.77 (IQR, 0.51–1.00) exams per year for the High-IS group and the Low-IS group, respectively. As shown in Fig. 2a, the High-IS group showed significantly worse BCSS compared with that of the Low-IS group [*p* < 0.001, hazard ratio (HR), 3.13; 95% CI 2.32–4.21]. Since more patients with advanced stages underwent frequent imaging studies, we stratified the patients by their tumor stage and observed consistent results (Fig. 2b–d, Table 2). Moreover, the statistically significant association between the surveillance intensity and survival was shown after stratification by patient’s age, hormonal receptor status, or HER2 overexpression status (Fig. 3a–f).

**Table 2:** Univariate and multivariable analyses for breast cancer-specific survival

Characteristics	Univariate analysis		Multivariable analysis ^a^	
	HR [95% CI]	*p* value	HR [95% CI]	*p* value
Surveillance intensity				
Low-intensity	Ref.	< 0.001	Ref.	0.263
High-intensity	3.13 [2.32–4.21]		1.27 [0.84–1.93]	
Institution volume				
High volume	Ref.	0.293	–	–
Low volume	1.17 [0.88–1.56]			
Age at operation (years)				
< 40	Ref.	0.022	Ref.	0.608
≥ 40	0.68 [0.49–0.95]		0.90 [0.60–1.36]	
BMI (kg/m^2^)				
< 25	Ref.	0.050	Ref.	0.095
≥ 25	1.33 [1.00–1.76]		1.33 [0.95–1.86]	
Anatomic stage ^b^				
I	Ref.	< 0.001	Ref.	< 0.001
II	3.54 [2.37–5.31]		2.50 [1.39–4.51]	
III	10.22 [6.79–15.38]		3.86 [1.98–7.50]	
Histologic grade				
I–II	Ref.	< 0.001	Ref.	0.002
III	2.91 [2.21–3.84]		1.81 [1.24–2.63]	
Lymphovascular invasion				
Present	Ref.	< 0.001	Ref.	< 0.001
Absent	0.37 [0.29–0.49]		0.46 [0.33–0.65]	
Hormone receptor status				
Positive	Ref.	< 0.001	Ref.	0.004
Negative	2.60 [2.01–3.35]		1.73 [1.19–2.52]	
HER2 receptor status				
Positive	Ref.	0.024	Ref.	0.026
Negative	0.72 [0.54–0.96]		0.45 [0.23–0.91]	
Ki-67 index ^c^				
Low	Ref.	< 0.001	Ref.	0.228
High	1.83 [1.40–2.39]		1.24 [0.87–1.77]	
Neoadjuvant chemotherapy				
Administered	Ref.	< 0.001	Ref.	< 0.001
Not administered	0.17 [0.13–0.23]		0.33 [0.21–0.53]	
Adjuvant chemotherapy				
Administered	Ref.	0.035	Ref.	0.306
Not administered	0.72 [0.53–0.98]		1.25 [0.82–1.91]	
Adjuvant radiotherapy				
Administered	Ref.	< 0.001	Ref.	< 0.001
Not administered	1.62 [1.22–2.15]		2.10 [1.48–2.98]	
HER2-targeted treatment				
Administered	Ref.	0.054	Ref.	0.009
Not administered	0.73 [0.53–1.01]		2.72 [1.28–5.79]	
Locoregional recurrence				
Absent	Ref.	< 0.001	Ref.	< 0.001
Present	9.59 [7.20–12.78]		6.26 [4.36–8.99]	

*HR* hazard ratio, *CI* confidence interval, *Ref* reference, *BMI* body mass index, *HER2* human epidermal growth factor receptor 2

Data on patients who underwent breast cancer surgery between January 2010 and December 2011 from the eleven hospitals in South Korea were retrospectively reviewed

^a^Variables with a *p* value < 0.10 in the univariate analysis were excluded in the multivariable analysis

^b^Stratified according to the American Joint Committee on Cancer (AJCC) eighth TNM anatomic stage

^c^Stratified according to each institution’s criteria

**Fig. 4:**
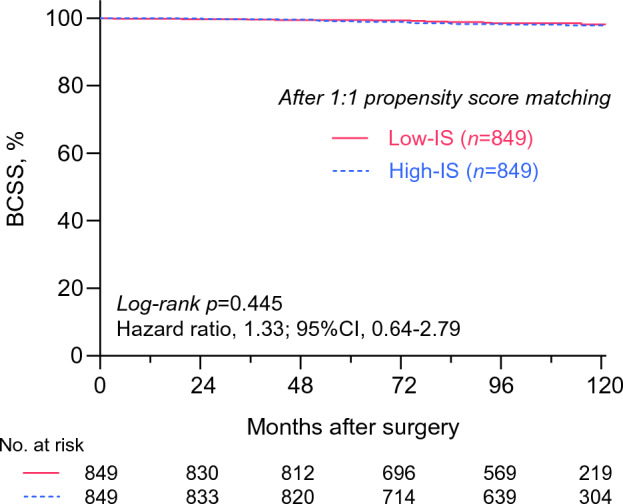
Kaplan–Meier curves showing breast cancer-specific survival after 1:1 propensity score matching. We performed 1:1 propensity score matching analysis to adjust for confounding effects of clinicopathologic variables of original cohort. A total of 1698 patients were yielded and there was no significant difference in BCSS according to the intensity of surveillance. *p* value was calculated by using the log-rank test, and the hazard ratio was calculated with univariate Cox-regression analysis. *BCCS* breast cancer-specific survival, *Low-IS* low-intensity surveillance, *High-IS* high-intensity surveillance, *CI* confidence interval

Multivariable Cox proportional hazards regression model of all patients showed that the screening intensity was not associated with BCSS after adjusting for relevant variables (*p* = 0.263, HR 1.27; 95% CI 0.84–1.93, Table 2). Moreover, the observed difference in the BCSS can be the result of uneven distribution of well-known risk factors between the two groups, since features associated with worse outcomes, such as younger age, advanced stages, or high histologic grade, were associated with the High-IS group (Table 3). Notably, the High-IS group was also significantly associated with high-volume institutions, molecular subtypes, and the use of systemic therapies. Thus, to adjust for these confounding effects, we performed a 1:1 propensity score matching analysis. Among the 4130 patients, a total of 1698 patients were included in the propensity score matching analysis with well-balanced variables (Table 3). In this analysis, we observed that the intensity of surveillance tests for metastasis was not related to significant survival differences (Fig. 4).

**Table 3:** Clinicopathological characteristics between the two groups before and after propensity score matching

	Before PSM			After PSM		
	Low-IS (*n* = 2065)	High-IS (*n* = 2065)	*p* value	Low-IS (*n* = 849)	High-IS (*n* = 849)	*p* value
Age at operation (years)						
< 40	232 (11.2%)	307 (14.9%)	0.001	76 (9.0%)	76 (9.0%)	1.000
≥ 40	1833 (88.8%)	1758 (85.1%)		773 (91.0%)	773 (91.0%)	
Institution volume						
High volume	1758 (85.1%)	1429 (69.2%)	< 0.001	717 (84.5%)	717 (84.5%)	1.000
Low volume	307 (14.9%)	636 (30.8%)		132 (15.5%)	132 (15.5%)	
Anatomic stage ^a^						
I	1053 (51.0%)	677 (32.8%)	< 0.001	334 (39.3%)	334 (39.3%)	1.000
II	827 (40.0%)	963 (46.6%)		427 (50.3%)	427 (50.3%)	
III	177 (8.6%)	417 (20.2%)		88 (10.4%)	88 (10.4%)	
Unknown	8 (0.4%)	8 (0.4%)		–	–	
Histologic grade						
I–II	1365 (66.1%)	1024 (49.6%)	< 0.001	525 (61.8%)	525 (61.8%)	1.000
III	604 (29.2%)	853 (41.3%)		324 (38.2%)	324 (38.2%)	
Unknown	96 (4.6%)	188 (9.1%)		–	–	
Lymphovascular invasion						
Present	590 (28.6%)	700 (33.9%)	< 0.001	277 (32.6%)	277 (32.6%)	1.000
Absent	1427 (69.1%)	1261 (61.1%)		572 (67.4%)	572 (67.4%)	
Unknown	48 (2.3%)	104 (5.0%)		–	–	
Molecular subtype						
HR+/HER2−	1407 (68.1%)	1204 (58.3%)	< 0.001	580 (68.3%)	580 (68.3%)	1.000
HR+/HER2+	202 (9.8%)	281 (13.6%)		78 (9.2%)	78 (9.2%)	
HR-/HER2+	149 (7.2%)	227 (11.0%)		51 (6.0%)	51 (6.0%)	
HR-/HER2−	298 (14.4%)	338 (16.4%)		140 (16.5%)	140 (16.5%)	
Unknown	9 (0.4%)	15 (0.7%)		–	–	
Ki-67 index ^b^						
High	797 (38.6%)	692 (33.5%)	0.030	316 (37.2%)	316 (37.2%)	1.000
Low	1173 (56.8%)	1176 (56.9%)		533 (62.8%)	533 (62.8%)	
Unknown	95 (4.6%)	197 (9.5%)		–	–	
Adjuvant or neoadjuvant chemotherapy						
Administered	1322 (64.0%)	1740 (84.3%)	< 0.001	683 (80.4%)	683 (80.4%)	1.000
Not administered	577 (27.9%)	296 (14.3%)		166 (19.6%)	166 (19.6%)	
Unknown	166 (8.0%)	29 (1.4%)		–	–	
Adjuvant radiotherapy						
Administered	1679 (81.3%)	1604 (77.7%)	< 0.001	722 (85.0%)	722 (85.0%)	1.000
Not administered	364 (17.6%)	460 (22.3%)		127 (15.0%)	127 (15.0%)	
Unknown	22 (1.1%)	1 (0.0%)		–	–	
Adjuvant hormonal treatment						
Administered	1588 (76.9%)	1487 (72.0%)	< 0.001	656 (77.3%)	656 (77.3%)	1.000
Not administered	462 (22.4%)	577 (27.9%)		193 (22.7%)	193 (22.7%)	
Unknown	15 (0.7%)	1 (0.0%)		–	–	
HER2-targeted treatment						
Administered	233 (11.3%)	386 (18.7%)	< 0.001	105 (12.4%)	105 (12.4%)	1.000
Not administered	1735 (84.0%)	1671 (80.9%)		744 (87.6%)	744 (87.6%)	
Unknown	97 (4.7%)	8 (0.4%)		–	–	
Locoregional recurrence						
Present	40 (1.9%)	135 (6.5%)	< 0.001	4 (0.5%)	4 (0.5%)	1.000
Absent	2025 (98.1%)	1930 (93.5%)		845 (99.5%)	845 (99.5%)	
Distant metastasis						
Absent	1989 (96.3%)	1840 (89.1%)	< 0.001	832 (98.0%)	832 (98.0%)	1.000
Bone metastasis	12 (0.6%)	57 (2.8%)		4 (0.5%)	4 (0.5%)	
Visceral single organ	27 (1.3%)	106 (5.1%)		4 (0.5%)	4 (0.5%)	
Multiple organs	37 (1.8%)	62 (3.0%)		9 (1.1%)	9 (1.1%)	

Number of patients (%)

Data on patients who underwent breast cancer surgery between January 2010 and December 2011 from the 11 hospitals in South Korea were retrospectively reviewed

*High-IS* high-intensity surveillance, *Low-IS* low-intensity surveillance, *PSM* propensity score matching, *HR* hormone receptor, *HER2* human epidermal growth factor receptor-2

^a^Stratified according to the American Joint Committee on Cancer (AJCC) eighth TNM anatomic stage

^b^Stratified according to each institution’s criteria

## Discussion

The present retrospective analysis of the 4130 breast cancer patients from 11 teaching hospitals in Korea shows the frequent use of imaging studies in breast cancer patients in real-world practice. Additionally, our data demonstrate that the use of intensive surveillance for breast cancer patients was not associated with an improved survival outcome despite the use of currently available therapeutic modalities.

Current guidelines that recommend against the use of routine imaging tests to detect distant metastasis in asymptomatic patients with breast cancer stemmed from the results of two randomized trials reported in 1994.^6–9^ The Interdisciplinary Group for Cancer Care Evaluation (GIVIO) randomized 1320 patients with breast cancer into the intensive surveillance group and the control group.^8^ The intensive surveillance group routinely conducted bone scan, liver sonography, chest x-ray, and laboratory tests, while the control group only conducted clinically indicated tests. With a median follow-up of 71.0 months, they reported that intensive surveillance had no significant impact on survival outcomes and quality of life. Del Turco et al. also randomized 1243 patients and showed that intensive surveillance was significantly associated with earlier detection of distant metastasis, while the overall survival was comparable between the two groups.^9^ Additionally, Kokko et al. prospectively randomized 472 patients who underwent surgery between 1991 and 1995, and reported that intensive surveillance tests were not associated with improved disease-free or overall survival but did increase surveillance costs by 2.2 times.^19^ However, it is important to note that these trials were conducted before the era of recent advancements in therapeutic regimens and highly sensitive imaging modalities.^20^

Jung et al. have shown that, among various postoperative imaging studies, only mammography showed prognostic implication based on the insurance reimbursement data for patients who were treated between 2002 and 2010.^21^ However, the limitation of this study was that the intrinsic nature of the insurance reimbursement data prohibited the investigators from collecting detailed data on the demographic and clinicopathologic features and the recurrence data. Furthermore, we have previously shown the lack of survival differences according to the intensity of metastasis surveillance before developing distant metastasis in 398 patients who were treated between 2000 and 2015 in a single institution.^22^ In the present study, we demonstrate that the use of frequent imaging tests to screen distant metastasis in patients with breast cancer was not related to improved BCSS based on a multi-institution dataset consisting of detailed clinicopathologic information and long-term follow-up. Our findings are particularly noteworthy that the patients included in the present study were treated with contemporary standard treatment that was unavailable during the pivotal trials conducted decades ago.

Our data also show that, despite the current guidelines, many physicians prescribe various imaging tests to detect distant metastasis in patients with breast cancer after their initial treatment for real-world clinical practice. This practice, which may vary among regions or institutions, can reflect the common belief that earlier detection of distant metastasis might improve the survival outcomes of breast cancer patients.^10–12,23–25^ Another reason for performing various imaging tests for metastasis surveillance is to provide reassurance for disease-free breast cancer patients.^26,27^ However, it is worthwhile to note that an intensive surveillance approach may also cause harms to the patients. Patients often experience anxiety and fear during their follow-up exams, and intensive surveillance programs result in an increased financial burden to the healthcare system.^19,24,28,29^ Most importantly, earlier detection of distant metastasis by intensive use of imaging studies can increase the duration of palliative treatment without meaningful survival gain, an effect known as the lead-time bias.^22,30,31^ Interestingly, our data demonstrate that the use of intensive metastasis surveillance resulted in significantly earlier detection of lung and bone metastasis but not of liver metastasis. Lung and bone metastasis in breast cancer are associated with more indolent clinical progression compared to the liver metastasis that shows poor post-metastasis survival similar to the brain or multiple organ metastasis.^32^ These findings indicate that patients who develop lung or bone metastasis can experience a significantly longer duration of the lead-time bias effect when they undergo intensive surveillance.

Moreover, early detection with higher sensitivity exams may enable accurate and timely treatment as numerous ongoing trials investigating adjuvant therapeutics have shown promising results.^13–16,33–35^ Thus, in an era of novel imaging modalities and treatment regimens, continuous investigations will be necessary to establish concrete evidence on whether intensive surveillance leads to prolonged survival or merely contributes to prolonged lead-time bias without providing any survival benefits.

The current study has several limitations. First, the nature of the retrospective study carries an inherent selection bias. As observed in our results, patients with more aggressive tumor features tended to undergo intensive screening exams. A large-scale randomized-controlled study is currently being conducted in Japan to determine the impact of intensive follow-up on survival outcomes, and we expect that the results of this study would provide a more definitive answer to this issue.^36^ Another limitation of this retrospective study was that we were not able to collect accurate information on metastasis-related symptoms. Symptomatic patients with more advanced stage of metastasis may have conducted intensive surveillance before diagnosis. Moreover, we could not determine whether the cause of death was directly owing to breast cancer or to complications from treatment, such as sepsis caused by an infection during chemotherapy. However, given the relatively low incidence of deaths from treatment complications in patients with breast cancer, it might have a minimal impact on our results.^37^ Second, we did not review in detail whether the imaging tests were conducted for screening of breast cancer metastasis or for other medical purposes. However, it is important to note that imaging tests performed for other medical diseases can also identify breast cancer metastasis. Thus, including all tests conducted for various diseases during the disease-free period would be appropriate for the purpose of our study. Third, as we excluded male patients with breast cancer from the study, our results may not fully represent the real-world data. Finally, although our dataset represents a relatively recent cohort for whom most of the contemporary adjuvant treatment options are provided, there are some novel therapeutic options that were not available, such as CDK4/6 inhibitors or pertuzumab.^16,33^ The implication of these recently developed therapeutic options in breast cancer with distant metastasis should be further explored in the future.

## Conclusions

In this multi-institutional retrospective study involving 4130 breast cancer patients who were treated between 2010 and 2011, the use of frequent imaging studies to detect distant metastasis resulted in earlier detection of distant metastasis, especially for lung and bone metastasis. However, intensive surveillance was not associated with prolonged BCSS.

## Supplementary Information

Below is the link to the electronic supplementary material.

Supplementary file1 (DOCX 462 KB)

## Data Availability

Jong-Ho Cheun had full access to all the data in the study and takes responsibility for the integrity of the data and the accuracy of the data analysis.
