# Supplementary material for: Surveillance for Distant Metastasis in Breast Cancer Patients Who Underwent Contemporary Management: A Report from the Korean Breast Cancer Society Survivor Research Group
Source: Ann Surg Oncol. 2024 Jul 5;31(10):6774–85. doi: 10.1245/s10434-024-15665-3 (PMC11413078; doi:10.1245/s10434-024-15665-3)
Supplement: Supplementary file 1 — Supplementary file1 (DOCX 462 KB) [file 10434_2024_15665_MOESM1_ESM.docx]

**eSupplemental material**

**Surveillance for distant metastasis in breast cancer patients who underwent contemporary management: a report from the Korean Breast Cancer Society Survivor Research Group**

**Table of contents**

Method of grouping patients into high-intensity surveillance and low-intensity surveillance groupeFigure 1

**eFigure 1. Method of grouping patients into high-intensity surveillance and low-intensity surveillance group**


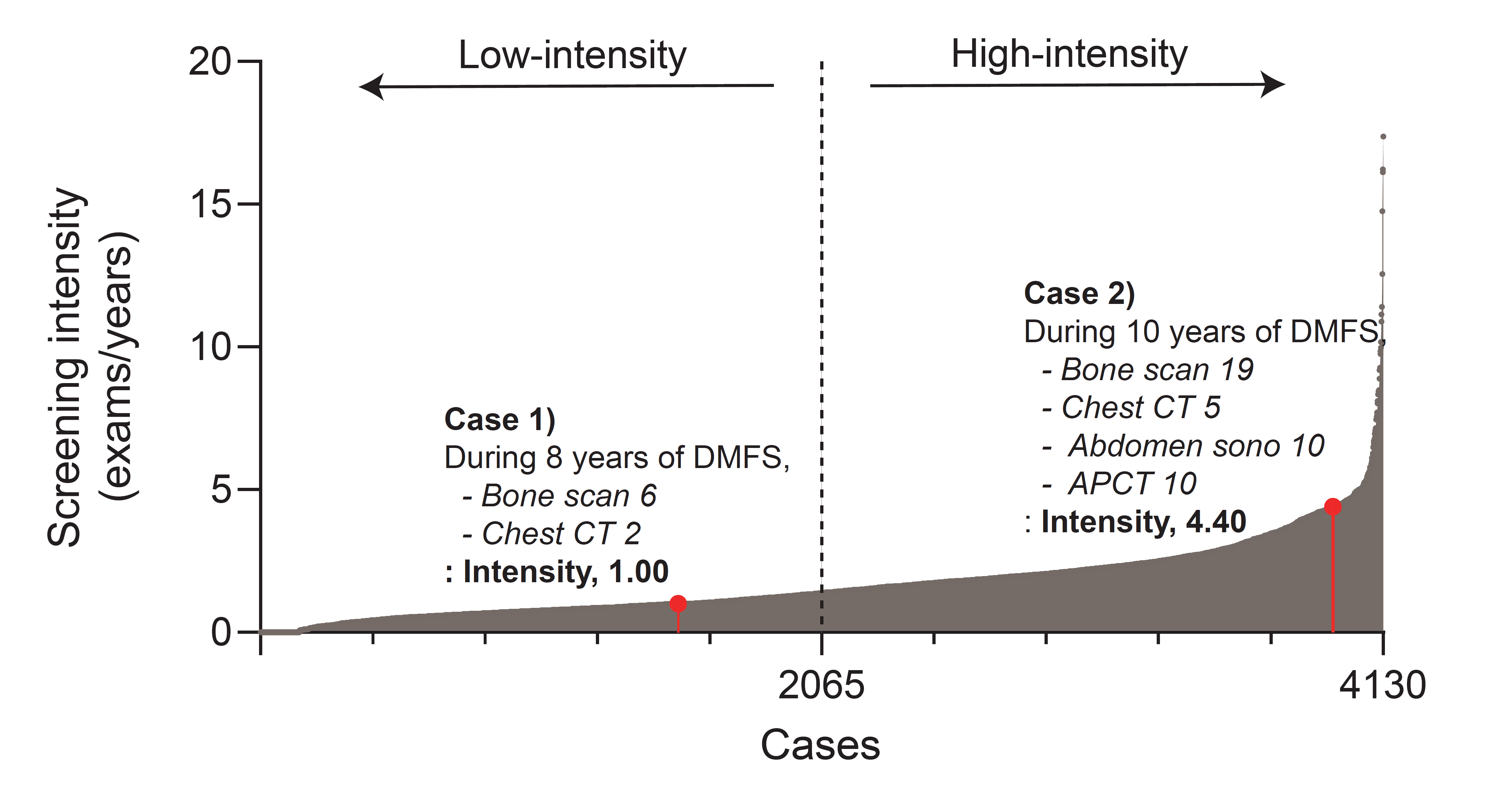


Patients were divided into two groups based on the median value of measured screening intensity, calculated by dividing the total number of exams by the DMFS interval. When the patient conducted a total of eight exams during eight years of DMFS, the intensity was 1.00 (Case 1). Meanwhile, when the patient performed 44 exams during ten years, the intensity was 4.40 (Case 2). As the median value of screening intensity of all patients was 1.38, Case 1 and Case 2 were grouped as the Low-intensity and High-intensity groups, respectively.

Abbreviations: DMFS, distant metastasis-free survival; CT, computed tomography; APCT, abdominal-pelvic computed tomography
